# Effect of Copolymer Latexes on Physicomechanical Properties of Mortar Containing High Volume Fly Ash as a Replacement Material of Cement

**DOI:** 10.1155/2014/670710

**Published:** 2014-08-31

**Authors:** El-Sayed Negim, Latipa Kozhamzharova, Yeligbayeva Gulzhakhan, Jamal Khatib, Lyazzat Bekbayeva, Craig Williams

**Affiliations:** ^1^Faculty of Science and Engineering, University of Wolverhampton, Wulfruna Street, Wolverhampton WV1 1LY, UK; ^2^Polymers and Pigments Department, National Research Centre, Dokki, Giza 12622, Egypt; ^3^Taraz State University Named After M.H. Dulati, 60 Tole Bi Street, Taraz 080000, Kazakhstan; ^4^Kazakh National Technical University Named After K.I. Satpayev, 22 Satpayev Street, Almaty 050013, Kazakhstan

## Abstract

This paper investigates the physicomechanical properties of mortar containing high volume of fly ash (FA) as partial replacement of cement in presence of copolymer latexes. Portland cement (PC) was partially replaced with 0, 10, 20, 30 50, and 60% FA. Copolymer latexes were used based on 2-hydroxyethyl acrylate (2-HEA) and 2-hydroxymethylacrylate (2-HEMA). Testing included workability, setting time, absorption, chemically combined water content, compressive strength, and scanning electron microscopy (SEM). The addition of FA to mortar as replacement of PC affected the physicomechanical properties of mortar. As the content of FA in the concrete increased, the setting times (initial and final) were elongated. The results obtained at 28 days of curing indicate that the maximum properties of mortar occur at around 30% FA. Beyond 30% FA the properties of mortar reduce and at 60% FA the properties of mortar are lower than those of the reference mortar without FA. However, the addition of polymer latexes into mortar containing FA improved most of the physicomechanical properties of mortar at all curing times. Compressive strength, combined water, and workability of mortar containing FA premixed with latexes are higher than those of mortar containing FA without latexes.

## 1. Introduction

Replacement cementing materials (RCMs) such as fly ash (FA), silica fume, and ground granulated blast furnace slag have been used in blended cement or in concrete in recent years more than in the past due to some benefits. These benefits of RCMs include energy saving and reduction in CO_2_, which is a major greenhouse gas causing global warming, emitted during cement production [1]. However, the percentage amount of RCMs in binder of cementitious system is limited, as it may adversely affect the mechanical properties if used beyond certain proportions but not necessary for the durability properties [2–8].

Number of studies of the effects of fly ashes on the behavior of cement pastes, mortars, and concretes were also carried out. In these studies a standard 30 wt.% replacement of cement by fly ash and a standard water/cement ratio of 0.5 were used. However, a number of researchers reported that 50% of fly ash is used as RCMs [9, 10]; class F and C fly ash are used in about 15–40% by mass of cementitious. Strength of the concretes in which fly ash is used instead of up to 30% of the cement is lower than Portland cement concretes at the early stages but the ultimate strength in the later years is higher. Andrzej and Joanna [11] reported that the content of conventional fly ash in polymer mortar affects positively their mechanical strengths and chemical resistance; however in a limited range exceeding the limit content values resulted in deterioration of properties. The 15% FA content in polymer concretes and mortars appeared to be proper value also when analyzing composites chemical resistance. Dosage varies with the reactivity of the ash and the desired effects on the concrete [12, 13].

During the last few decades, there are increasing interests in the use of both RCMs and chemical admixtures in order to improve the physical and mechanical properties of concrete [14–25]. The addition of fly ash reduces the dosage of chemical admixture needed to maintain adequate fluidity in grouts.

Negim et al. [26, 27] prepared hydrophilic copolymer latexes based on 2-hydroxyethyl acrylate (2-HEA) and 2-hydroxyethyl methacrylate (2-HEMA) using macroradical initiator technique. Different ratios of acrylic monomers were designed to investigate the effects of the hydrophilic copolymers on physicomechanical properties of Portland cement (PC) pastes and mortar. The results showed that the addition of copolymer latexes improved the properties of mortar more than those of PC. The work was further extended to include the application of the obtained copolymer latexes to modify physicomechanical properties of mortar containing varying content of flay ash as replacement cement material.

## 2. Experimental

### 2.1. Synthesis and Characterization of Copolymer Latexes

The preparation of copolymer latexes and the methods of analysis (^1^H NMR, FT-IR, mass spectroscopy, and viscosity) have been previously described [26]. The mix proportion of monomers in the copolymer and basic properties of synthesized copolymer are shown in Table 1.

### 2.2. Cement and Fine Aggregate

The ingredients of mixes were cement, fly ash (FA), water, and sand. The cement that has been used is Portland cement (PC). PC and FA are conforming to BS EN 197-1, 2011 [28] and EN 450 [29], respectively. The chemical composition of PC and FA is shown in Table 2. The mineralogical composition of the PC sample is C_3_S: 58.79%, *β*-C_2_S: 17.68%, C_3_A: 8.08%, and C_4_AF: 9.72% as shown in Table 3. Sand (sizes: 0.21–0.53 nm) was used as the fine aggregate and is free from organic or clay-like materials. The fine aggregate used complied with class M of BS 882, 2004 [30].

### 2.3. Mixing Proportions

Eleven mortar mixes were used to examine the influence of copolymer latexes with different ratio of 2-HEA and 2-HEMA on the properties of mortar containing different amounts of fly ash (FA) as cement replacement. Details of mixes are given in Table 4. The reference mortar mix (M0) had a proportion of 1 (PC): 3 (sand) and did not include FA. In mixes M10–M60, PC was partially replaced with 10%, 20%, 30%, 50%, and 60% FA (by mass), respectively. The water to binder ratio for all mixes was maintained constant at 0.50. The binder consists of PC and FA. A constant dosage of copolymer latex of 1% by mass of binder was used for all mixes.

### 2.4. Tests

The prepared copolymer was added to mixing water and then added gradually to 300 g of the dry mortar in order to determine setting time using Vicat apparatus [31]. Workability test using the flow table was conducted as per BS 1881: part 105 [32]. Mortar specimens of dimensions 70 mm × 70 mm × 70 mm were cast in steel moulds and were prepared. The specimens were manually agitated for 2 minutes and then on a vibrator for another 2 minutes to assure the complete removal of air bubbles and voids and to produce homogenous pastes. The moulds were kept in a humidity chamber at 100% R. H and a constant room temperature overnight and then demoulded and cured under water for 1, 3, 7, and 28 days before testing. Testing included compressive strength, water absorption, and combined water. The compressive strength was determined according to BS EN 12390-3 : 2002 [33] whereas the water absorption and combined water tests were according to BS 1881-122 : 2011 [34].

## 3. Results and Discussion

### 3.1. Structure of Copolymers

**Scheme 1:**
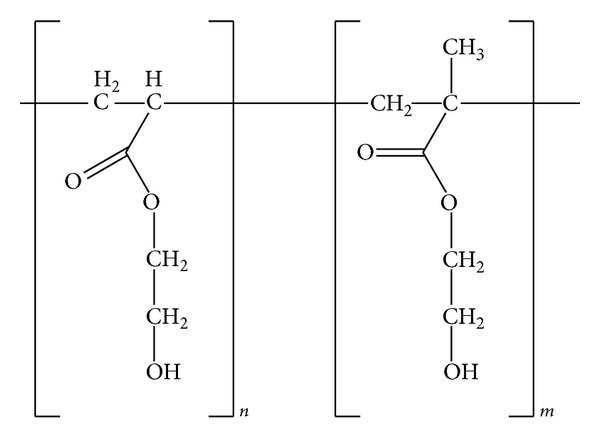
Structure of P(2-HEA-co-2-HEMA) [26].

The structure of the copolymer latexes based on 2-hydroxyethyl acrylate (2-HEA) and 2-hydroxyethyl methacrylate (2-HEMA) is shown in Scheme 1. The copolymer latexes were synthesized with different ratios (95 : 05, 90 : 10, and 85 : 15, resp.,) using azobisisobutyronitrile as free radical initiator. The properties of the prepared copolymer latexes have been previously reported by Negim et al. [26]. The results showed that physicomechanical properties of the copolymer were increased by increasing the ratio of 2-HEMA in the copolymer latexes.

### 3.2. Workability

**Figure 1:**
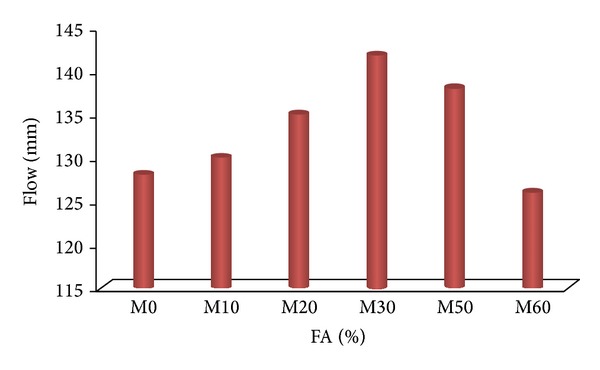
The effect of FA on the workability of mortar mixes without latexes.

Figure 1 shows the flow of mortar with varying amounts of FA as replacement cement material (RCM) compared with reference mortar M0 (i.e., 0% FA) at constant water/binder ratio (0.5) without copolymer latexes. Generally, using the fly ash in the mortar increases the workability of the fresh mortar [35, 36]. When the replacement level of fly ash (FA) increases from 10 to 30%, the workability of mortar increases from 128 to 142 mm. Freshly mixed mortar is generally more workable when a portion of the cementitious material is fly ash, because of the spherical shape of fly ash particles as in the study reported in [37]. When the replacement levels of FA are increased from 50 to 60%, the workability decreased from 138 to 126 mm, due to the more water absorbed by the material, that is, the negative effect of FA, as expected and in agreement with previously reported results by Liu et al. [37].

**Figure 2:**
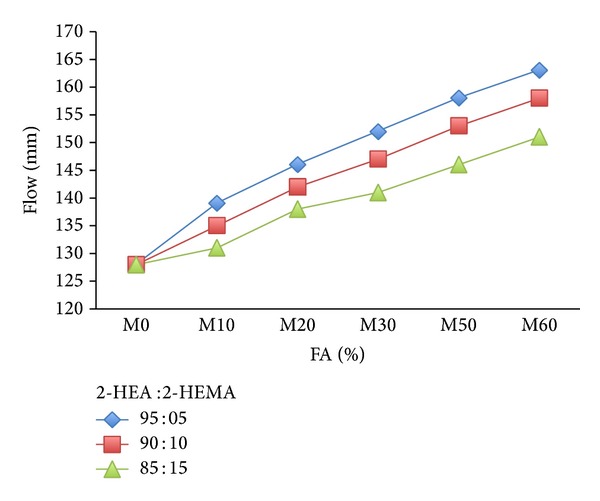
The effect of FA on the workability of mortar mixes in presence of latexes.

Figure 2 shows a sharp increase in flow of mortar with increasing amount of fly ash as RCMs in presence of copolymer latexes at constant water/binder ratio (0.5). However, an increase in the 2-HEA ratios in copolymer latexes increases the workability of the mortar. Mortar mixed with 60% (FA) and copolymer containing 95% (2-HEA) gave the highest flow with 163 mm while mortar mixed with 10% (FA) and copolymer containing 85% (2-HEA) showed a flow measurement of 151 mm. The increase in flow of mortar premixed with FA as RCMs in presence of copolymer latexes due to the ball bearing action of polymer particles improved the fluidity of the concrete [38]. It is well established that the use of FA in mortar reduces the water demand for a given workability. Therefore, mortar containing FA and acrylic latexes will cause an increase in workability at constant water to binder ratio due to the ability of acrylic polymer to be adsorbed on binder particles, thus providing dispersing effect on the mortar.

### 3.3. Setting Time

**Figure 3:**
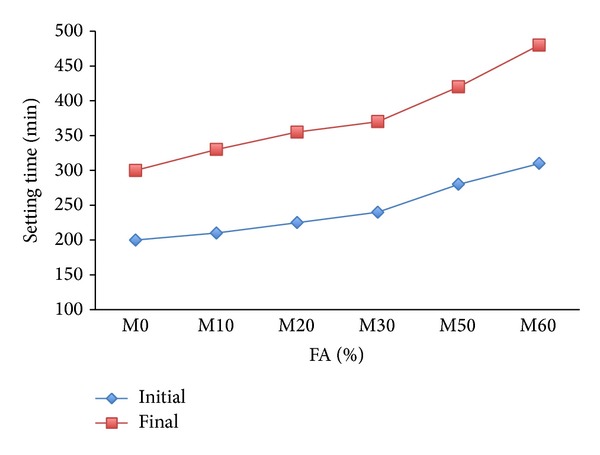
The effect of FA on the setting time of mortar mixes without latexes.

The initial and final setting times of mortar containing different amount of fly ash as RCMs without copolymer latexes are present in Figure 3. As a result of the increased FA content in mortar, initial and final setting time are increased. This is attributed to the slow pozzolanic reactions of fly ash and the decreased amount of cement (60% fly ash) as in the study reported in [39].

**Figure 4:**
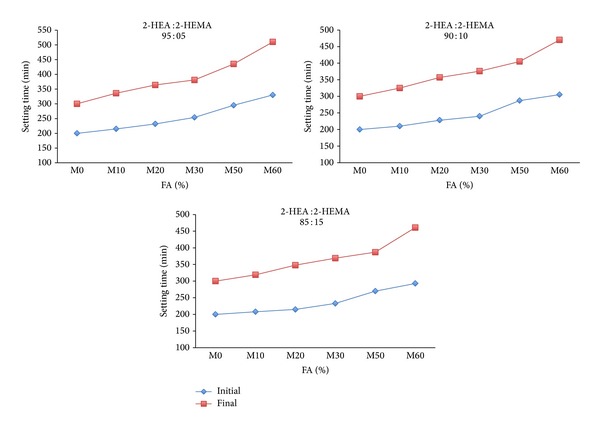
The effect of FA on the setting time of mortar mixes in presence of latexes.

Effects of 1% copolymer latexes with varying feeds of 2-HEA and 2-HEMA on setting times of mortar containing FA in comparison to corresponding reference mortar M0 are shown in Figure 4. From results, it can be seen that the initial and final setting time of mortar premixed with copolymer latex containing 95% 2-HEA were longer than those of the corresponding references mortar M0, while the incorporation of copolymer latex containing 85% 2-HEA reduced the initial and final setting times of mortar with varying amounts of FA. It is well known that the setting of fresh concrete is affected by the kind and time of addition of organic admixtures [40]. In addition, the retarding of setting time of mortar is due to the dispersion effects provided by the copolymer latexes and the mineral admixtures on the cement particles. Furthermore, it can be seen from Figures 3 and 4 that copolymer latexes combined with FA in mortar are a much more effective retarder than FA.

### 3.4. Water Absorption

**Figure 5:**
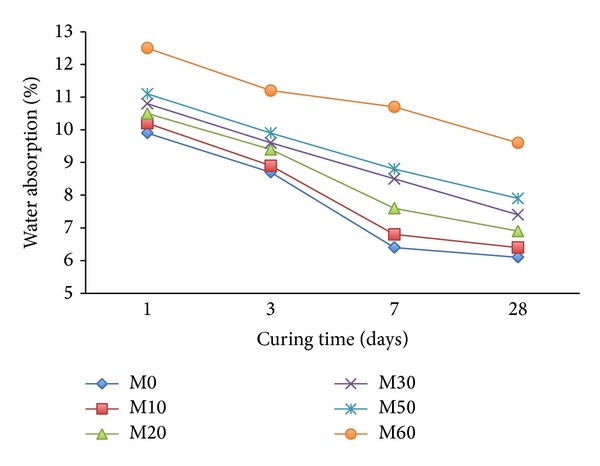
The effect of FA on water absorption of mortar mixes without latexes.

**Figure 6:**
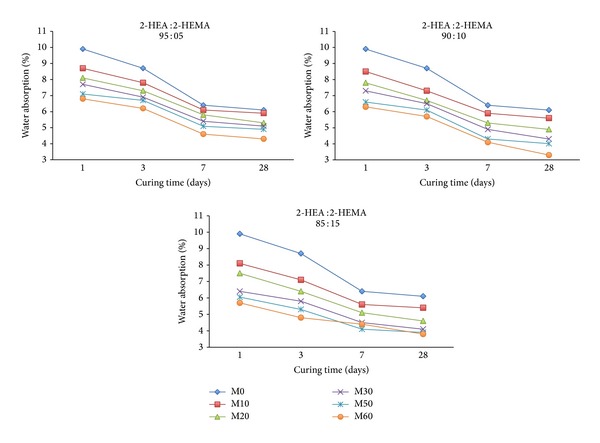
The effect of FA on water absorption of mortar mixes in presence of latexes.

Results of water absorption of mortar containing varying amounts of FA with and without copolymer latexes are given in Figures 5 and 6. It can be seen in the Figure 5 that with the increase of content of FA in mortar mixes the water absorption increases. Mixes containing 50 and 60% FA as replacement of cement show higher water uptake and higher water absorption. All the mortars with FA showed low water absorption with increasing the curing time from 1 day to 28 days. This is attributed to the reduction in pore size of cement from the reaction with FA prior to 28 days after mixing [41, 42]. The water absorption of the mortars depends upon the porosity and the pore size of cement. On the other hand, the incorporation of copolymer latexes improves the water absorption properties of mortar containing FA as shown in Figure 6. The water absorption of mortar with copolymer latexes is lower by 4% compared to mortar without the copolymer latexes. However, water absorption of mortar decreases with increasing the ratio of 2-HEA in the copolymer latexes and FA content in the mortar; this is attributed to a very good water-resistant bond between the polymer and the binder components. This results from the reduction of permeable voids with increasing 2-HEA. Generally, the absorption values for all mixes in Figure 6 are lower than those reported in another investigation [43].

### 3.5. Compressive Strength

**Figure 7:**
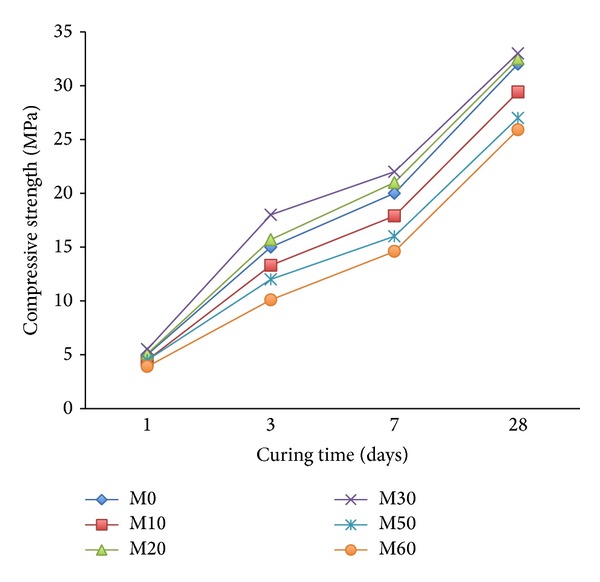
The effect of FA on compressive strength of mortar mixes without latexes.

**Figure 8:**
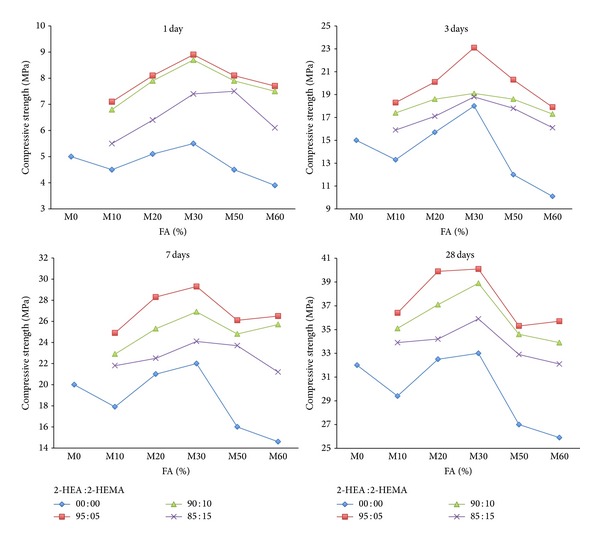
The effect of FA on compressive strength of mortar mixes in presence of latexes.

The compressive strength of the mortar containing varying amounts of FA and those premixed with the copolymers latexes are given in Figures 7 and 8. It is generally obvious that the compressive strength of mortar mixes increases slightly with curing time up to seven days but sharply increases for up to 28 days. This is mainly due to the fact that at early ages fly ash exhibits very little cementing value. At later ages when liberated lime resulting from hydration of cement reacts with fly ash and contributes considerable strength to the concrete. It can be seen in Figure 7 that there is a decrease in compressive strength as the amount of FA is increased. For example, the strength for the control mix (M0, 0% FA at 28-day age) is 32.6 MPa and this drops down to 27 and 25.9 MPa for the mortar mix containing 50 and 60% FA as a replacement of cement. The maximum compressive strength seems to occur at about a replacement level of 20–30% FA, which seems to agree with the results of a previous investigation [44]. The results confirm that the compressive strength of fly ash mortar depends on the amount of fly ash [2, 4, 45, 46]. Use of copolymer latexes along with FA and cement helps to provide specific properties of mortar like increased long term strength as shown in Figure 8. However, an increase in the ratio of 2-HEA in the copolymer latexes causes an increase in compressive strength of the mortar mixes containing varying amounts of FA. Mortar containing 20 and 30% FA as replacement of cement shows substantially higher compressive strength than the other one. This is principally due to the gradual increase in the process of polymerization or crystallization resulting from an increase in the branching of the used copolymeric materials. The same behavior was reported by authors in [27, 47] when they studied the effect of acrylic polymer on physicomechanical properties of Portland cement mortar.

### 3.6. Chemically Combined Water Content

**Figure 9:**
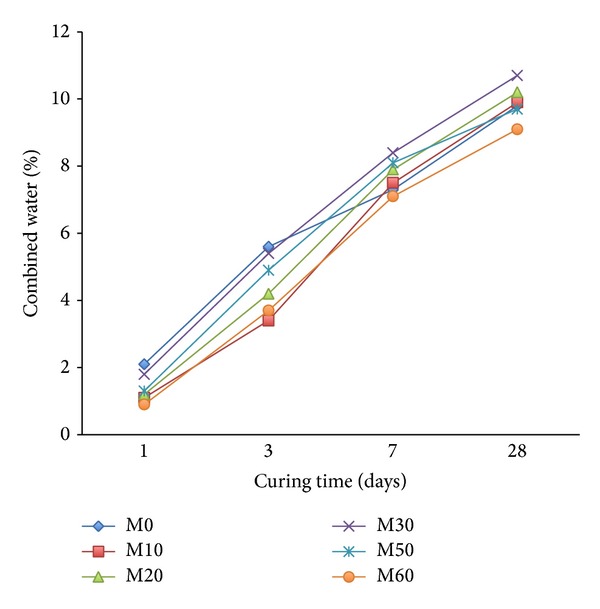
The effect of FA on combined water content of mortar mixes without latexes.

The combined water contents of mortars mixes containing different amounts of FA without copolymer latexes are present in Figure 9. The results showed that the combined water content of all mixes increased with curing time. This was mainly attributed to the gradual and continuous formation of hydration products resulting from the hydration of the main phases of cement, particularly C_3_S and *β*-C_2_S [48].

The combined water content of mortar mixes with varying amounts of FA is lower than the reference mortar M0 during the early age of hydration up to 7 days and then becomes higher during the later ages up to 28 days. This may be attributed to the fact that the pozzolanic reaction between FA and cement grains was slow at early ages. At 28 days, the combined water of mixes increased from 9.9% to 10.7% as the FA content increased from 10 to 30 wt.% and decreased from 9.7 to 9.1% as FA content increased from 50 to 60% (Figure 9).

**Figure 10:**
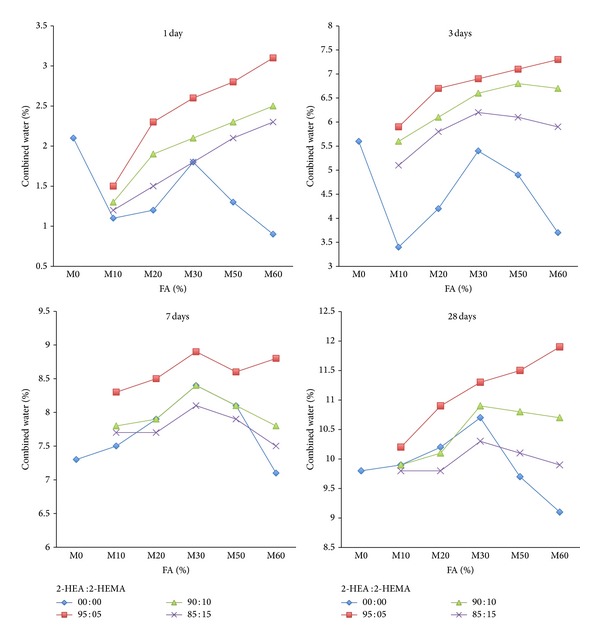
The effect of FA on combined water content of mortar mixes in presence of latexes.

On the other hand, the influence of copolymer latexes on the combined water of mortar mixes containing varying amounts of FA is given in Figure 10. Generally, the combined water content of all mortar mixes gradually increased with curing time. Furthermore, the combined water contents of mortar containing varying contents of FA premixed with copolymer tend to be higher than reference mortar mix M0 at all curing periods. It is attributed to the fact that the cement hydration process generally precedes the polymer film formation process by the coalescence of polymer particles in adduct polymer. Furthermore, the combined water content of the mixes with the FA increases with the increase in the ratio of 2-HEA in the copolymer latexes. It is proved that an admixture having a functional group that influences the morphology of hydrate is produced [41]. Furthermore, the combined water content of mortar premixed with copolymer latexes is higher than that mortar without latexes.

### 3.7. SEM

**Figure 11:**
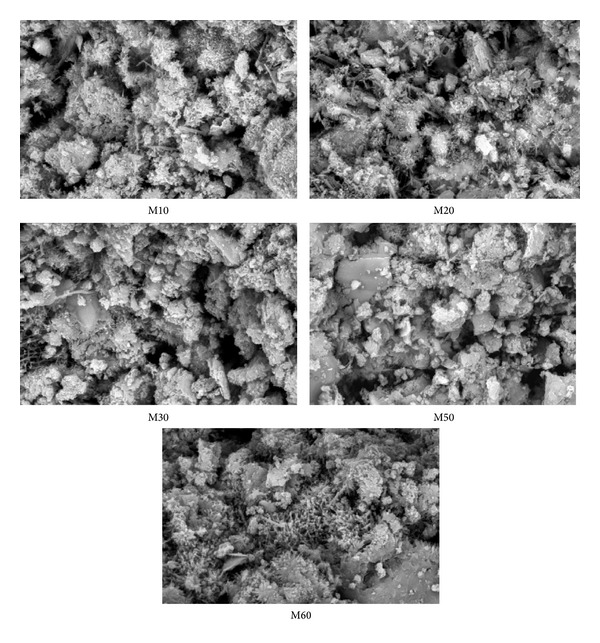
Scanning electron micrographs of mortar containing different content of FA without copolymer latexes.

Figure 11 shows the surface of mortar containing varying amounts of FA at 10, 20, 30, 50, and 60% as replacement of cement, respectively. From Figure 11, it can be seen that there are voids in mortar-FA mixes. However, as the FA content increases from 10 to 30% the number and size of voids increase. This confirms the improvement in the flow of mortar containing 10 and 30% FA. When the replacement levels of FA are increased from 50 to 60%, the workability decreased because of reducing the size of the void and also the void, which becomes uniform in shape.

**Figure 12:**
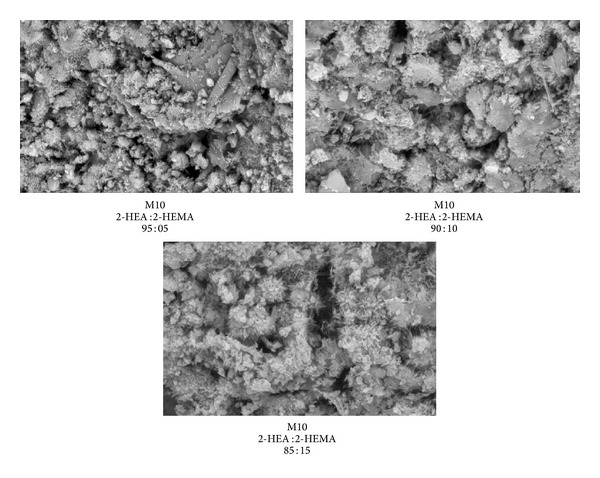
Scanning electron micrographs of mortar containing 10% FA in presence of latexes.

Figure 12 shows the surface of mortar containing 10% FA premixed with copolymer latexes. There is progressive decrease in the size of pores in the surface of the mortar due to vibration of the water molecules and formation of polymer films. As the 2-HEA ratio increases in the copolymer latexes, the size and number of pores are decreased. This confirms the improvement of compressive strength of mortar containing FA premixed with copolymer latexes, which is attributed to partial filling of the pore system leading to a more dense structure of the hardened cement paste with FA. Similar findings based on Na-phenol sulfonate formaldehyde, Na-polystyrene sulfonate, and Na-*β*-naphthol sulfonate formaldehyde condensates have been reported [49]. From Figures 11 and 12, it can be observed that pores generated from mortar containing FA without copolymer latexes are higher than those premixed with copolymer latexes. This confirms the higher water absorption in mortar containing FA without copolymer latexes.

## 4. Conclusions

The physicomechanical properties of mortar containing FA as replacement cement material with and without copolymer latexes were investigated and can be summarized as follows.

1. Flow of mortar mixed with varied amount of FA as partial replacement of PC in presence of copolymer latexes is higher than that of mortar containing FA, due to the fact that a large quantity of air is entrained in mortar because of an action of the polymer contained in mortar.
2. The setting times (initial and final) were prolonged, that is, especially with the 50% and 60% FA in presence of copolymer latexes.
3. Mortar premixed with 60% FA in presence of copolymer exhibited lower water absorption than that of another content of FA and reference mortar (M0).
4. There is an increase in the chemically combined water content and strength of mortar containing FA in presence of copolymer latexes compared with those without latexes at all curing ages.
5. Replacing cement with up to at least 30% FA causes increase in strength compared to the control mix. However, using more than 60% FA as partial cement replacement does not cause further increase in strength compared with the control.
6. The SEM micrographs of mortar confirm the improvement of compressive strength of mortar containing FA premixed with copolymer latexes, which is attributed to partial filling of the pore system leading to a more dense structure of the hardened cement paste with FA.
7. The copolymer latexes, which were used in the mortar containing FA, played an important role in the specific characteristics of the mortar and allowed higher replacements of cement with FA (up 60%).
8. Mixing of mortar with high percentage of FA as RCMs in the presence of copolymer latexes improves most of the specific characteristics of mortar and reduces the cost of concrete.

## Figures and Tables

**Table 1:** Properties of P(2-HEA-co-2-HEMA) [26].

2-HEA	2-HEMA	*T*_*g*_^a^	*T*_*g*_^b^	Appearance	pH
95	05	−12.22	−14.22	Liquid	7.0
90	10	−9.37	−10.3		
85	15	−6.47	−8.1		

^a^Predicted using Fox equation.

^b^Measured using DSC.

**Table 2:** Chemical composition of Portland cement and fly ash.

Composition	PC	FA
SiO_2_ (%)	20.1	50.5
Al_2_O_3 _(%)	4.9	24.7
Fe_2_O_3 _(%)	2.4	7.4
CaO (%)	65	2.6
SO_3 _(%)	2.3	0.8
MgO (%)	3.1	1.5
Insoluble residue (%)	1.9	—
Loss on ignition (%)	2.0	—
Lime saturated factor	0.85	—
Specific surface area (m^2^/kg)	290	356

**Table 3:** The mineralogical composition of Portland cement.

Composition (%)	PC
C_3_S	58.79
*β*-C_2_S	17.68
Fe_2_O_3_	2.4
C_3_A	8.08
C_4_AF	9.72

**Table 4:** Composition of binder.

Binder (%)		
Mix number	PC	FA
M0	100	0
M10	90	10
M20	80	20
M30	70	30
M50	50	50
M60	40	60
